# Rural-urban differences on the rates and factors associated with early initiation of breastfeeding in Nigeria: further analysis of the Nigeria demographic and health survey, 2013

**DOI:** 10.1186/s13006-017-0141-x

**Published:** 2017-12-29

**Authors:** Emmanuel Olorunleke Adewuyi, Yun Zhao, Vishnu Khanal, Asa Auta, Lydia Babatunde Bulndi

**Affiliations:** 1 Statistical and Genomic Epidemiology Laboratory, Institute of Health and Biomedical Innovation, Queensland University of Technology, Brisbane, Australia; 2 Federal Ministry of Defense, 2 Division Hospital, Adekunle Fajuyi Cantonment, Ibadan, Oyo State, Nigeria; 3 Department of Epidemiology and Biostatistics, School of Public Health, Curtin University, Bentley Campus, Perth, Australia; 4 Nepal Development Society, Bharatpur, Chitwan Nepal; 5 School of Pharmacy and Biomedical Sciences, University of Central Lancashire, Preston, UK; 6 Mashiah Foundation Hospital, Jos, Plateau State, Nigeria

**Keywords:** Breastfeeding initiation, Determinants, Infants feeding, Mothers, Rural-urban Nigeria

## Abstract

**Background:**

This study investigates and compares the rates and factors associated with early initiation of breastfeeding (EIBF) within one hour of birth in rural and urban Nigeria.

**Methods:**

Data from the 2013 Nigeria Demographic and Health Survey (NDHS) were analyzed. The rates of EIBF were reported using frequency tabulation. Associated factors were examined using Chi-Square test and further assessed on multivariable logistic regression analysis.

**Results:**

The rates of EIBF were 30.8% (95% confidence interval [CI] 29.0, 32.6) and 41.9% (95% CI 39.6, 44.3) in rural and urban residences, respectively (*p* < 0.001). The North-Central region had the highest EIBF rates both in rural (43.5%) and urban (63.5%) residences. Greater odds of EIBF in rural residence were significantly associated with higher birth order (Adjusted Odds Ratio [AOR] 1.29, 95% CI 1.10, 1.60), large birth size (AOR 1.33, 95% CI 1.10, 1.60), and health facility delivery (AOR 1.46, 95% CI 1.23, 1.72). Rural mothers in the rich wealth index, not working and whose husbands obtained at least a secondary school education had significantly higher odds of early initiation of breastfeeding. Regardless of residence, greater odds of EIBF were significantly associated with non-cesarean delivery (Rural AOR 3.50, 95% CI 1.84, 6.62; Urban AOR 2.48, 95% CI 1.60, 3.80) and living in North-Central (Rural AOR 1.84, 95% CI 1.34, 2.52; Urban AOR 4.40, 95% CI 3.15, 6.15) region. Also, higher odds of EIBF were significantly associated with living in North-East (Rural AOR 1.48, 95% CI 1.05, 2.08; Urban AOR 3.50, 95% CI 2.55, 4.83), South-South (Rural AOR 1.51, 95% CI 1.11, 2.10; Urban AOR 2.84, 95% CI 2.03, 3.97) and North-West (Urban residence only AOR 2.08, 95% CI 1.54, 2.80) regions.

**Conclusions:**

Rural-urban differences in the rates and factors associated with EIBF exist in Nigeria with rural residence having significantly lower rates. Intervention efforts which address the risk factors identified in this study may contribute to improved EIBF rates. Efforts need to prioritize rural mothers generally, (particularly, those in rural North-West region) as well as mothers in urban South-West region of Nigeria.

## Background

Early initiation of breastfeeding (EIBF) is the practice of introducing breast milk to newborns in which baby is placed skin-to-skin with the mother within the first hour of delivery, as recommended by the World Health Organization (WHO) [1]. Being the first and the most important step to optimal breastfeeding (early initiation, exclusive for the first six months and then complementary breastfeeding for the first two years of life), EIBF remains one effective means of promoting the health and survival status of infants and mothers [2]. Timely initiation of breastfeeding within the first hour of birth means neonates are introduced to colostrum (‘first milk’) which confers numerous benefits including active and passive immunity against a wide range of pathogenic diseases [1, 2]. It also reduces postpartum bleeding in mothers, as well as protects against the leading causes of neonatal mortality such as respiratory tract infections (pneumonia, in particular), diarrhea, and neonatal sepsis [2–5]. Maternal-child bonding, longer duration and greater breastfeeding success are some of the beneficial effects of EIBF [6]. Moreover, a convincing body of evidence reveals that EIBF reduces neonatal mortality by approximately 22% in Ghana [3] and 19% in Nepal [7]. Despite its numerous benefits and the WHO recommendation, the rate of breastfeeding initiation within the first hour of delivery is poor in many countries around the world [2].

In Nigeria, breastfeeding ('any breastfeeding') is universally practiced, however, the rate of EIBF is low and declining [8, 9]. Evidence from the national households surveys in Nigeria indicates that the rate of EIBF increased from 31.9% in 2003 to 38.4% in 2008 but decreased sharply to 33.2% in 2013 [9]. These rates are lower than those of similar developing countries like Ghana, the Gambia and Malawi at 46%, 48% and 56%, respectively [10]. Studies have shown that a range of factors; socioeconomic, community, health, maternal and individual, may influence the rate of initiation of breastfeeding [11, 12]. In view of this premise, it is imperative to understand factors associated with EIBF in Nigeria. Regrettably, nationally representative studies on the rates and factors associated with breastfeeding initiation are limited in Nigeria.

Two recent and nationally representative studies have investigated the determinants of EIBF in Nigeria. Using the pooled (for the whole Nigerian population) 2013 Nigeria Demographic and Health Survey (NDHS), a study found a significant association between EIBF and place of delivery, mode of delivery, parity, birth size, maternal occupation, wealth index, and rural-urban residence [12]. Similarly, another study examined the trends and determinants of EIBF from 1990 to 2008 and reported maternal age at child’s birth, mother’s and father’s education level, parity, residence, region, antenatal visit, mode and place of delivery as being significantly associated with EIBF [13]. However, all the studies to date only provided estimates based on the national average (overall Nigerian population) of EIBF and are limited, in that the within-population variations such as the rural-urban differences were not assessed. In other words, they were based only on the pooled datasets and not disaggregated by rural-urban residence. Using pooled datasets or national average of estimates may mask the within population differences [14]. Thus, a gap in knowledge exists on how the rates and factors associated with EIBF differ between rural and urban residences in Nigeria. The present study aims to bridge this knowledge gap by disaggregating the most recent NDHS dataset along rural-urban residence thereby providing evidence-based, context-specific knowledge for addressing the challenge of low rates of EIBF in Nigeria.

The rural-urban stratification approach adopted in our study is in line with the growing consensus on using high quality disaggregated studies as an evidence-based method to bridge access/survival/equity gaps across socioeconomic and/or geographic divides [15, 16]. Furthermore, the method agrees with the recent WHO’s framework for monitoring progress towards Universal Health Coverage (UHC) which states: ‘all measures should be disaggregated by socioeconomic and demographic strata’ for a better assessment of equity in intervention coverage among other factors [14]. The approach may help capture context-specific factors which might not be possible with the use of ‘one-size-fits-all’ method of pooled datasets [16–18]. Given the beneficial effects of EIBF and the critical need for accelerated reduction in neonatal mortality, especially, in light of the ambitious targets for UHC (inscribed in the 2030 agenda for sustainable development) [16, 17], this study provides further evidence for target-specific interventions aimed at improving EIBF rates in Nigeria.

## Methods

Dataset from the nationally representative NDHS, 2013, was analyzed in this study. NDHS is a public domain dataset available freely online (www.dhsprogram.com) with permission from ICF, International USA. A stratified three-stage cluster sampling design was used for sample collection in the survey and the design consisted of 904 clusters: 532 in rural residence and 372 in urban areas [9]. Interviewer-administered structured questionnaires were used for data collection and a total of 40,320 representative households were selected for the survey [9]. However, only 38,522 (22,663 in rural areas and 15,859 in urban areas), out of the 38,904 households occupied at field work time were successfully interviewed [9]. To reduce possible recall bias, data analyzed in this study were restricted to a total of 11,851 mothers who provided information on breastfeeding initiation on their last live childbirth within two years preceding the 2013 NDHS [9, 12]. A comprehensive report on the setting, sampling design, questionnaires and sampling frame for the 2013 NDHS has previously been published [9].

### Study factors

The outcome variable for this study was EIBF, defined as introducing breastfeeding to newborns within the first hour of delivery in line with the WHO recommendation [1]. Responses to the question on ‘when child was put to breast’ following delivery, in the 2013 NDHS [9], was re-categorized as ‘within one hour’ (early initiation, coded as ‘1’) and ‘beyond one hour’ (late initiation, coded as ‘0’) for use in logistic regression analyses. We selected independent variables based on the objective of this study and the review of previous studies [11–13] with consideration of the information available in the 2013 NDHS. The variables were broadly categorized into three groups; socioeconomic, biodemographic and health/support factors, in line with practice in previous studies [17–19].

Socioeconomic variables assessed in this study included mother’s and father’s education level (secondary/higher, primary and none*), mother’s occupation [re-categorized as not working (not working, households and domestic works), agriculture (paid and unpaid agricultural works) and paid works* (office, business, clerical, both skilled and unskilled manual) [11, 20], and wealth index. Wealth index, a proxy for household’s socioeconomic status, was derived in the survey through the principal component analysis of available households’ assets. Based on the similarities between the five categories captured in the 2013 NDSH, the variable was re-categorized into three in the present study as follows: rich = richer + richest, middle = middle, and poor* = poorer + poorest.” [18].

Biodemographic variables assessed were birth type (single* and multiple), mother’s age (re-categorized as: < 20*, 20–34, ≥ 35 years), sex of child (male and female*), mother’s religion (re-categorized as: Christianity, Islam* and Traditional/other) and birth size (re-categorized as follows large = very large + larger than average, average = average and small* = very small + smaller than average). ‘Birth size’ describes mothers’ perception of the size of their babies at birth. The variable was used as a substitute for ‘birthweight’ given that information on birthweight was substantially missing in the 2013 NDHS, a common feature of most population-based surveys in developing countries like Nigeria [9, 21]. There is evidence, however, that birth size estimates are usually closely related to the values of birthweight [22], thus, justifying the substitutionary use of the variable.

Other biodemographic factors included the region of residence (categorized according to the geo-political zones in Nigeria: North-Central, North-East, North-West, South-East, South-South and South-West*), mother’s marital status (never married, formerly married and currently married*) and birth order (1*, 2–3, and ≥4). Health seeking/support factors, including antenatal attendance (none*, 1–3 and ≥4), mode of delivery (cesarean section* and non-cesarean section vaginal) as well as the place of delivery (home* and health facility) were equally assessed in this study. These variables and their categorization compares well with those of previous studies in Nigeria and internationally [11, 12, 17–19]. (Note: * is the reference category used in analyses).

### Statistical analysis

The rate (in %) of EIBF alongside its 95% CI were obtained against each aforementioned explanatory variables using frequency tabulation. Chi-Square test was performed and *p* - values were reported to assess the unadjusted association between EIBF and the various explanatory variables by comparing the differences in the rate of EIBF between variable categories. To evaluate the adjusted relationship between EIBF and the explanatory variables, multivariable binary logistic regression analyses were carried out, accounting for the effects of all other explanatory variables included in the models.

To ensure that no important explanatory factors were missed, variables with *p* ≤ 0.20 in the Chi-Square test were selected for inclusion in the initial multivariable regression model in line with practice in previous studies [18, 23]. This cut-off point was chosen following a critical appraisal of evidence in the literature [24–26]. A backward elimination regression analysis was then performed to obtain the final parsimonious model, which only retained explanatory variables significantly associated with EIBF at 5% level (*p* - value <0.05). Adjusted odds ratios in the final parsimonious models together with their 95% CI and *p* - values were reported. To reduce possible statistical errors, analyses were double-checked, and all variables that satisfied inclusion criterion were included in our models. Also, our backward elimination modelling was tested by including potential confounders. All these analysis procedures were carried out separately for data disaggregated by rural-urban residence. All data management and analyses were performed using the Statistical Package for Social Sciences (SPSS), version 21. The sample weight and the multistage cluster design of the 2013 NDHS were accounted for using the Complex Sample Analysis method [27]. Where appropriate, the term ‘residence’ and ‘areas’ were used interchangeably in this study.

## Results

### Rates of EIBF in rural and urban residence

**Fig. 1:**
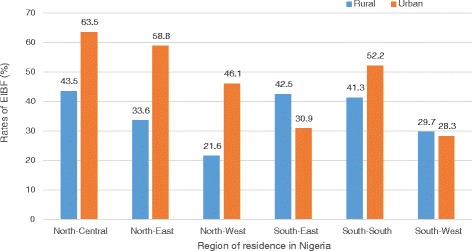
Regional differences in the rates of Early Breastfeeding Initiation in Nigeria by rural-urban residence

The demographic details of the sample, together with the percentage of mothers who fed breast milk to their newborns within the first hour of delivery are shown in Table 1. The rates of EIBF were 30.8% (95% CI 29.0, 32.6) and 41.9% (95% CI 39.6, 44.3) in rural and urban residence (*p* < 0.001), respectively (Table 1). Regionally, the highest rates of EIBF occurred in the North-Central region both in urban (63.5%, *p* < 0.001) and rural (43.5%, *p* < 0.001) residences. On the other hand, the South-West (28.3%, *p* < 0.001) and the North-West (21.6%, *p* < 0.001) regions, had the lowest rates of EIBF in urban and rural residences, respectively (Fig. 1). Compared to their counterparts with no education (26.1%), the rate of EIBF in rural residence was higher among mothers who had at least a secondary school education (41.9%, *p* < 0.001). Conversely, in urban residence, mothers with no education had a higher rate of EIBF (51.2%) than their counterparts who had a secondary/higher education (39.1%, *p* < 0.001).

**Table 1:** Sample characteristics and rates of early initiation of breastfeeding by rural and urban residence in Nigeria, NDHS 2013

Factors	Rural residence			Urban residence		
	N^a^ (%)^b^	Rate of EIBF^b^		N^a^ (%)^b^	Rate of EIBF^b^	
		% (95 %CI)	*p*-value		% (95 %CI)	*p*-value
Socioeconomic factors						
Mother’s education level			<0.001			<0.001
Secondary/higher	1837 (20.6)	41.9 (38.7, 45.1)		2345 (59.7)	39.1 (36.4, 41.9)	
Primary	1514 (17.2)	34.5 (31.5, 37.6)		776 (19.4)	40.6 (36.1, 45.3)	
None	4592 (62.2)	26.1 (23.9, 28.5)		787 (20.9)	51.2 (46.2, 56.2)	
Mother’s Occupation			<0.001			0.011
Paid work	4092 (52.6)	28.3 (26.1, 30.5)		2624 (68.0)	40.5 (38.0, 43.1)	
Agriculture	1140 (13.6)	37.5 (33.2, 41.9)		201 (4.4)	38.2 (31.3, 45.7)	
Not working	2657 (33.8)	32.3 (29.5, 35.1)		1059 (27.6)	46.1 (42.1, 50.1)	
Father’s education level			<0.001			0.009
Secondary/higher	2540 (30.3)	39.8 (37.3, 42.4)		2536 (66.8)	41.4 (38.6, 44.2)	
Primary	1456 (18.4)	33.4 (30.1, 36.9)		674 (17.8)	39.0 (34.2, 44.0)	
None	3667 (51.3)	24.7 (22.3, 27.1)		558 (15.4)	50.4 (44.2, 56.6)	
Wealth index			<0.001			0.408
Rich	1303 (14.3)	44.2 (40.2, 48.2)		2808 (73.4)	42.2 (39.6, 44.9)	
Middle	1682 (20.4)	35.5 (32.3, 38.9)		686 (16.2)	43.5 (37.4, 49.8)	
Poor	4958 (65.3)	26.4 (24.3, 28.7)		414 (10.4)	37.5 (31.0, 44.5)	
Biodemographic factors						
Mother’s marital status			0.539			0.046
Never married	203 (2.1)	27.3 (20.7, 35.0)		115 (2.6)	28.8 (19.7, 40.0)	
Formerly married	169 (1.9)	34.0 (25.1, 44.1)		100 (2.4)	38.9 (28.8, 49.9)	
Currently married	7571 (96.0)	30.8 (29.0, 32.7)		3693 (95.0)	42.4 (40.0, 44.8)	
Mother’s age			0.007			0.135
≥ 35	1654 (20.2)	31.3 (28.3, 34.5)		837 (21.6)	45.7 (41.3, 50.1)	
20–34 years	5498 (69.2)	31.6 (29.6, 33.6)		2900 (73.8)	40.8 (38.2, 43.5)	
< 20 years	791 (10.6)	24.8 (21.1, 28.9)		171 (4.6)	42.5 (33.8, 51.7)	
Mother’s religion			<0.001			<0.001
Christianity	2783 (30.1)	40.0 (37.0, 43.0)		2057 (50.3)	35.8 (32.9, 38.9)	
Traditional/other	111 (1.7)	34.7 (22.3, 48.1)		45 (1.0)	40.9 (25.4, 58.5)	
Islam	5049 (68.2)	26.7 (24.5, 28.9)		1806 (48.7)	48.3 (44.8, 51.7)	
Sex of child			0.323			0.320
Male	4042 (50.3)	31.3 (29.2, 33.5)		1979 (50.0)	41.0 (38.0, 44.0)	
Female	3901 (49.7)	30.3 (28.3, 32.4)		1929 (50.0)	42.9 (39.9, 46.0)	
Birth order			0.187			0.143
1	1457 (18.7)	28.7 (25.8, 31.9)		874 (22.4)	38.3 (34.1, 42.7)	
2–3	2387 (30.3)	32.1 (29.5, 34.8)		1420 (35.9)	42.6 (38.9, 46.3)	
≥ 4	4099 (51.0)	30.8 (28.7, 33.0)		1614 (41.7)	43.4 (40.2, 46.6)	
Birth size			<0.001			0.015
Large	3403 (43.4)	33.7 (31.3, 36.2)		1801 (45.2)	45.1 (41.6, 48.6)	
Average	3164 (39.8)	30.2 (27.6, 32.9)		1612 (42.2)	38.8 (35.6, 42.1)	
Small	1329 (16.8)	24.9 (22.0, 28.1)		482 (12.6)	40.8 (35.6, 46.2)	
Birth type			0.570			0.043
Multiple	131 (1.8)	28.3 (20.5, 37.6)		66 (1.6)	27.3 (16.4, 41.8)	
Single	7812 (98.2)	30.9 (29.1, 32.7)		3842 (98.4)	42.2 (39.8, 44.6)	
Region of residence			<0.001			< 0.001
North-Central	1207 (16.2)	43.5 (39.2, 47.9)		532 (9.3)	63.5 (57.3, 69.3)	
North-East	1911 (19.9)	33.6 (29.9, 37.6)		509 (12.2)	58.8 (52.4, 64.8)	
North-West	3001 (44.2)	21.6 (19.0, 24.4)		698 (22.4)	46.1 (40.6, 51.7)	
South-East	383 (4.4)	42.5 (34.4, 51.0)		693 17.7)	30.9 (26.1, 36.2)	
South-South	994 (9.5)	41.3 (36.4, 46.3)		437 (9.3)	52.2 (46.0, 58.3)	
South-West	447 (5.8)	29.7 (24.6, 35.4)		1039 (29.1)	28.3 (24.6, 32.2)	
Health-seeking/support factors						
Antenatal visit			<0.001			
≥ 4	3209 (39.3)	36.5 (34.0, 39.0)		2934 (77.3)	40.1 (37.6, 42.8)	0.027
1–3	1136 (14.4)	31.7 (27.9, 35.5)		437 (12.0)	48.0 (42.3, 53.7)	
None	3446 (46.3)	25.0 (22.5, 27.7)		391 (10.7)	47.6 (39.6, 55.7)	
Mode of delivery			0.032			<0.001
Non-CS	7844 (98.9)	30.9 (29.1, 32.8)		3664 (95.9)	43.0 (40.5, 45.5)	
CS	87 (1.1)	18.5 (10.7, 29.9)		176 (4.1)	24.1 (17.1, 32.8)	
Place of delivery			<0.001			0.761
Health facility	1942 (23.1)	42.4 (39.3, 45.5)		2546 (63.5)	41.7 (38.9, 44.5)	
Home	5984 (76.9)	27.3 (25.4, 29.3)		1355 (36.5)	42.4 (38.5, 46.4)	

Significance at 5% level. ^a^unweighted count. ^b^weighted percentage, *NDHS* Nigeria demographic and health survey

There was no statistically significant difference in the rates of EIBF between home (42.4%) and health facility (41.7%, *p* = 0.761) delivery in urban residence. However, in rural areas, delivery in a health facility was significantly associated with higher rates of EIBF (42.4%) than home delivery (27.3%, *p* < 0.001). Similarly, rich households in rural areas had significantly higher rate of EIBF (44.2%) than their counterparts in poor households (26.4%, *p* < 0.001). In urban areas, there was no statistically significant difference in the rate of EIBF between the rich (42.2%) and poor households (37.5%, *p* = 0.408).

Furthermore, rural mothers with at least four antenatal attendance had higher EIBF rates (36.5%) than those who had no antenatal attendance (25.0%, *p* < 0.001). The reverse was the case in the urban residence where the rates of EIBF was significantly higher among mothers who had attended no or 1–3 times antenatal care compared to their counterparts with ≥4 attendances (*p* = 0.027). Mothers who were delivered by cesarean section had lower rates of EIBF than their counterparts with non-cesarean delivery, regardless of living areas (Table 1). In rural areas, mothers professing Christianity had higher EIBF rates (40.0%) than their Muslim counterparts (26.7%, *p* < 0.001). On the other hand, Muslim mothers in urban areas had higher EIBF rates (48.3%) than Christian mothers (35.8%, *p* < 0.001).

### Factors associated with EIBF in rural and urban Nigeria

Table 2 presents the results of the multivariable regression analyses. For rural residence, three socioeconomic factors, wealth index, mother’s occupation and husband educational level, were significantly associated with EIBF. Similarly, three biodemographic (birth order, birth size and region of residence), as well as two health/support factors (place and mode of delivery), were found to be significantly associated with EIBF in the residence. Specifically, the odds of EIBF was 35% higher among rural mothers in rich households compared to their poor counterparts (AOR 1.35, 95% CI 1.06, 1.71), and 35% greater among mothers who were not working or those engaged in households/domestic works relative to their counterparts in paid jobs (AOR 1.35, 95% CI 1.15, 1.60). Mothers whose husband obtained at least a secondary school education had 28% increased odds of early initiation of breastfeeding (AOR 1.28, 95% CI 1.07, 1.57). Similarly, rural mothers of birth order ≥4 were 29% more likely to breastfeed within their first hour of delivery compared to those of birth order of one (AOR 1.29, 95% CI 1.10, 1.60). Further, the odds of EIBF was increased significantly among rural mothers living in the North-Central (AOR 1.84, 95% CI 1.34, 2.52), North-East (AOR 1.48, 95% CI 1.05, 2.08), and South-South (AOR 1.51, 95% CI 1.11, 2.10) regions. Also, the odds of EIBF were 33% and 46% significantly higher among mothers who had delivered a large sized baby and in a health facility, respectively (Table 2). Lastly, in the rural residence, the odds of EIBF were 3.5-fold higher among mothers with a non-cesarean delivery compared to those with a cesarean section (AOR 3.50, 95% CI 1.84, 6.62).

**Table 2:** Factors associated with early initiation of breastfeeding by rural-urban residence in Nigeria, NDHS 2013

Factors	Rural Residence			Urban Residence		
	AOR	95% CI	*p*-value	AOR	95% CI	*p*-value
Wealth index				–	–	–
Rich	1.35	1.06, 1.71	0.017	–	–	–
Average	1.11	0.94, 1.35	0.316			
Poor	1.00	Reference	–	–	–	–
Mother’s occupation				–	–	–
Not working	1.35	1.15, 1.60	< 0.001	–	–	–
Agriculture	1.05	0.82, 1.30	0.706			
Paid work	1.00	Reference	–	–	–	–
Husband’s education level				–	–	–
Secondary/higher	1.28	1.07, 1.57	0.010	–	–	–
Primary	1.20	0.99, 1.49	0.068			
None	1.00	Reference	–	–	–	–
Birth order				–	–	–
≥ 4	1.29	1.10, 1.60	0.003	–	–	–
2–3	1.14	0.94, 1.40	0.148			
1	1.00	Reference	–	–	–	–
Birth size				–	–	–
Large	1.33	1.10, 1.60	0.003			
Average	1.22	1.02, 1.46	0.032	–	–	–
Small	1.00	Reference	–	–	–	–
Region of residence						
North-Central	1.84	1.34, 2.52	< 0.001	4.40	3.15, 6.15	< 0.001
North-East	1.48	1.05, 2.08	0.027	3.50	2.55, 4.83	< 0.001
North-West	0.83	0.60, 1.20	0.340	2.08	1.54, 2.80	< 0.001
South-East	1.34	0.84, 2.13	0.224	1.12	0.82, 1.51	0.490
South-South	1.51	1.11, 2.10	0.010	2.84	2.03, 3.97	< 0.001
South-West	1.00	Reference	–	1.00	Reference	–
Place of delivery				–	–	–
Health facility	1.46	1.23, 1.72	< 0.001	–	–	–
Home	1.00	Reference	–	–	–	–
Mode of delivery						
Non-CS	3.50	1.84, 6.62	< 0.001	2.48	1.60, 3.80	< 0.001
CS	1.00	Reference	–	1.00	Reference	–

Significance at 5% level, *CS* Cesarean section, *AOR* Adjusted odds ratio, *NDHS* Nigeria Demographic and Health Survey

Factors adjusted for in rural residence: Mother’s education level, mother’s occupation, father’s education level, wealth index, mother’s age, mother’s religion, birth order, birth size, region of residence, antenatal visit, mode of delivery and place of delivery

Factors adjusted for in urban residence: Mother’s education level, mother’s occupation, father’s education level, marital status, mother’s age, mother’s religion, birth order, birth size, birth type, region of residence, antenatal visit and mode of delivery

In urban residence, no socioeconomic factor was significantly associated with EIBF. However, one biodemographic factor (region of residence) and one health/support factor (mode of delivery) attained statistical significance (Table 2). Compared to the South-West region, urban mothers living in the North-Central (AOR 4.40, 95% CI 3.15, 6.15), North-East (AOR 3.50, 95% CI 2.55, 4.83), North-West (AOR 2.08, 95% CI 1.54, 2.80), and South-South (AOR 2.84, 95% CI 2.03, 3.97) regions had increased odds of EIBF. As the case in rural residence, urban mothers who had a non-cesarean delivery had 2.5-fold increased odds of EIBF compared to those who had undergone a cesarean section (AOR 2.48, 95% CI 1.60, 3.80).

## Discussion

We assessed the rates and factors associated with early breastfeeding initiation in rural and urban Nigeria. Previous Nigerian studies [12, 13] have focused on the whole nation using pooled datasets (for the whole population). We have gone a step further to focus on the rural and urban residences in the country by analyzing disaggregated datasets. Our findings indicate that the rates of timely initiation of breastfeeding and the associated factors differ between rural and urban residences in Nigeria. For instance, a study has investigated the determinants of EIBF for the overall Nigerian population using the pooled 2013 NDHS [12]. The factors reported to be associated with EIBF by these authors; place of delivery, mode of delivery, parity, birth size, maternal occupation, and wealth index were mainly relevant in rural residence when we disaggregated the data by rural-urban residence. This highlights the importance of data disaggregation method in identifying population-specific factors for EIBF.

The finding that urban residence had significantly higher EIBF rates than rural residence is not surprising, and, several factors may have contributed to such difference. First, cultural practices/beliefs such as discarding colostrum because it ‘is dirty/rusty’ may contribute to lower rates of EIBF and this would expectedly be prevalent in rural than urban residence in Nigeria [28]. Second, access to and utilization of healthcare facility may facilitate the practice of early breastfeeding initiation, and rural residents in Nigeria are disproportionately disadvantaged in these regards [29, 30]. In the present study, for instance, mothers in urban residence had nearly two-fold greater antenatal attendance (of at least 4 times) and approximately three-fold higher prevalence of health facility delivery. Antenatal attendance and health facility delivery provide great avenues for information on the importance of EIBF. Hence urban women would more likely have acquired better breastfeeding knowledge, and, consequently, had higher EIBF rates than their counterparts in rural areas. Nevertheless, EIBF rates were generally low whether in rural or urban Nigeria (less than 50–89% deemed as good, and 90–100% considered as being very good by the WHO) [6]. This finding suggests the need for improved EIBF rates in both rural and urban Nigeria. However, given that rural residence had comparatively lower rates, greater attention is unarguably needed in the residence.

Consistent with previous Nigerian studies (overall Nigerian population only) [12, 13], our study found cesarean delivery to be significantly associated with decreased odds of EIBF both in rural and urban residence. This finding may be linked with the rigor, stress and exhaustion that often go with cesarean deliveries including the effects/complications of anesthesia [31]. The time-lapse between delivery, the repair of surgical incisions and recovery/responsiveness following cesarean section may contribute to late breastfeeding initiation. Also, given that most women are averse to cesarean delivery in Nigeria [32] and the fact that the obstetric intervention is usually performed as the last option in life-threatening conditions [32], the occurrence of complications is likely and such may further contribute to the risk of late breastfeeding initiation. Mothers who had undergone cesarean section may be less likely to introduce their newborns (which may also have difficulty sucking) to breastfeeding within the recommended one hour after birth. However, it is noteworthy that with suitable guidance and appropriate support, early initiation of breastfeeding is possible even among mothers who had undergone cesarean section [31].

Our study reveals that the rates and the odds of EIBF were significantly higher in the North-Central region whether in rural or urban residence. On the other hand, the North-West and the South-West regions had the lowest rates of EIBF in rural and urban residences, respectively. Following multivariable analysis, the odds of EIBF was significantly higher in the North-Central, North-East, and South-South regions relative to the South-West both in rural and urban residence. These findings agree to some extent with those of previous studies for the overall Nigerian population [12, 13]. However, the reason for these regional variations in EIBF rates and odds is not clear. A likely explanation, nonetheless, would be the impact of sociocultural practices/beliefs. For instance, in a community in South-West region of the country, the acceptable breastfeeding norms were prelacteal feedings of water, herbal infusions and ritual fluids [28]. Consequently, newborns in the said community were normally introduced to water and complementary feeding almost immediately after delivery [28]. Similarly, a study in the North-West region reported that colostrum was considered unfit for consumption in neonates because it was ‘dirty’ and ‘rusty’ [33]. These and similar sociocultural practices/beliefs may have contributed to the differences in rates and the odds of EIBF observed in the present study.

Notably, the association between EIBF and the region of residence was generally stronger in urban compared to rural residence. For instance, while mothers in both rural and urban North-Central region had greater odds of EIBF, the odds were over two-fold higher for urban mothers. This finding is similarly true for mothers in the North-East and the South-South regions indicating that urban mothers in the named regions had greater odds of initiating their newborns into breastfeeding within the recommended one hour after birth timeframe than their rural counterparts. The reason for this result may be linked with rural-urban disparities in breastfeeding knowledge, access to−/utilization of maternal cares services and socio-cultural practices in Nigeria [28–30]. Context-specific interventions are, therefore, needed to bridge these rural-urban differences in the country.

Wealth index attained statistical significance in the multivariable analysis, albeit in rural residence only. This finding is consistent with our Chi-Square test results where wealth index categories exhibited a strong and significant association with EIBF rates in rural residence. Contrariwise, wealth index categories showed only a negligible and nonsignificant difference in EIBF rates in urban residence suggesting that the rates of EIBF were similar among the rich, the middle class as well as the poor in urban Nigeria. The finding in urban areas clearly recommends breastfeeding as a simple, cost-effective intervention suitable for- and practicable by all irrespective of socioeconomic status (wealth index category). Conversely, in rural residence, both the rates and the odds of EIBF were significantly higher in wealthy compared to poor households. A previous study using the pooled 2013 NDHS has reported a similar result for the whole Nigerian population [12]. However, a rural-urban disaggregation (as carried out in our study) indicates that the result applies mainly to rural residence in Nigeria [12]. Thus, poor households in rural Nigeria would require greater priority for improved EIBF rates.

A critical review of the literature, however, reveals mixed results in respect of the association between EIBF and wealth index. For instance, a study conducted in Indonesia found that mothers classed in the rich wealth index had increased odds of late initiation of breastfeeding compared to those in poor wealth category [34]. Furthermore, wealthy mothers in Pacific, East Asia, Middle East, and North Africa regions have been reported to have greater chances of later breastfeeding initiation than their poor counterparts [34, 35]. These mixed findings may be explained by factors ranging from differences in educational attainment, and thus the level of breastfeeding knowledge to the marketing of breast milk substitutes and the purchasing power of the rich or subsidy for the poor [36]. Experience, attitudes, nature of work and the belief of mothers may equally contribute in some ways to the observed results.

Similar to the results for wealth index, health facility delivery was associated with increased odds and higher rates of EIBF than home delivery, but in rural residence only. This result may be credited to the effectiveness of Baby Friendly Hospital Initiative in rural Nigeria and our finding compares well with those of studies (pooled/national average only) in Nigeria [12], Nepal [11] and Indonesia [34]. However, the EIBF rate of 42.4% found in health facility for rural residence falls below the 90–100% recommended by the WHO suggesting the need to step up this initiative for a greater improvement in breastfeeding initiation rates in the residence. Besides, with only 23.1% (as found in the present study), the prevalence of health facility delivery in rural Nigeria remains unacceptably low. Future interventions would need to take cognizance of these findings for a holistic program/intervention design. In urban Nigeria, the rates of EIBF were nearly same for both home and health facility deliveries, showing no statistical difference, hence the factor did not make a selection for multivariable modelling. These results coupled with those of wealth index support our earlier position that urban residents in Nigeria possibly had better knowledge on the benefits of EIBF, hence the likely support from health facility delivery and the effects of higher socioeconomic status did not confer any appreciable advantage. Intervention(s)/practice(s) which may be responsible for these findings in urban residence need(s) to be investigated and replicated in rural Nigeria.

Our finding reveals that mothers in rural residence whose husbands had at least a secondary school education were more likely to initiate their newborns into breastfeeding within one hour of birth. Consistently, studies have shown that husband’s support is the most important influence on breastfeeding initiation and continuation decisions [37, 38]. Hence, it is logical to anticipate that husbands with at least a secondary school education would have better breastfeeding knowledge and, thus, are able to support their spouse/partners for early breastfeeding initiation decision.

Furthermore, in rural residence only, mothers who were not working or those who reported being engaged in domestic/household works had greater odds of EIBF compared to their counterparts engaged in paid employments (office, business, clerical, as well as both skilled and unskilled manual). A similar result has been reported for the overall Nepalese population [11], and two reasons may explain the current finding. First, mothers in paid jobs are more likely to have better access to financial resources and thus more susceptible to the practice of formula feeding which in turn may contribute to the late initiation of breastfeeding. Second, cesarean delivery is known to contribute to late breastfeeding initiation and mothers in paid jobs may opt for the mode of delivery, given their financial capability to afford it [9, 11]. However, there have been mixed reports in respect of the association between mothers’ occupation and EIBF. For example, a study in Bangladesh found no association [39] while a prospective study in the United States found that mothers in professional jobs had higher odds of early breastfeeding initiation [40]. Future studies would need to further investigate the relationship between maternal occupation and EIBF.

Our findings further indicate that in rural residence, babies perceived as being small in size at the time of delivery or belonging to the first birth order had decreased odds of EIBF. Small sized babies may require special care owing to premature delivery and immaturity, thus, chances of late breastfeeding initiation may be higher among them compared to their large-sized counterparts. In the same vein, studies have shown that both the intention to breastfeed and early breastfeeding initiation is associated with previous breastfeeding experience [41]. Hence, findings in respect of the association of EIBF with birth size and parity in rural Nigeria agree with the literature [12, 41]. In urban residence, birth order and birth size did not attain statistical significance following adjustment for other variables in the multivariable logistic regression analysis. The finding is not surprising for ‘birth order’ given the result of our Chi-Square test that showed no significant difference in the rates of EIBF for the variable. Interestingly, the position expressed here may not hold strictly true in rural residence as ‘birth order’ which lacked statistical significance in the Chi-Square test became significant in the multivariable analysis justifying the criterion set (*p* ≤ 0.20) for model building in our study. The finding for ‘birth size’ in urban areas may be due to the masking effect of the ‘region of residence’ and ‘caesarean delivery’ which were overwhelmingly significant in the residence.

### Strengths and limitations

Our study presents a snapshot of the rates and factors associated with early breastfeeding initiation in rural and urban Nigeria. National representativeness, high response rate, application of complex sample statistics in all analyses (to adjust for sample weights and cluster design of the survey) and low missing data are some of the strengths of this study. Others include large sample size and the use of rural-urban data disaggregation method. Nevertheless, this study is limited in that the cross-sectional design of the survey does not allow the causal relationship to be estimated. Also, data were self-reported, collected retrospectively and so prone to social desirability and recall biases. However, restricting our study to data in the two years preceding the 2013 NDHS reduces the chances of recall bias. To the best of our knowledge, this is the first study to investigate rural-urban differences in EIBF rates and associated factors using nationally representative data in Nigeria.

## Conclusions

This study reveals the rural-urban differences in the rates and factors associated with EIBF in Nigeria. Findings indicate that efforts are needed for improved EIBF rates both in rural and urban residences, although greater attention is evidently required in rural areas in the country. Whether in rural or urban residence, it is imperative to address late breastfeeding initiation associated with cesarean delivery using appropriate guidance and supports. Also, findings suggest the need to prioritize mothers in urban South-West as well as those in the rural North-West regions for context-specific interventions including addressing sociocultural practices that may negatively impact on EIBF.

In rural residence, our study recommends improved health facility delivery and nutritional support to address late breastfeeding initiation associated with small birth size. Breastfeeding initiation support for primiparous mothers, and increased breastfeeding awareness/support for mothers in poor households are equally required in rural Nigeria. A multi-dimensional/sectorial approach including media campaign using appropriate behavior change communication models, the long-term approach of acquiring secondary/higher education, community mobilization and comprehensive training for health workers are implementable interventions. These would need to give a special consideration for rural residence in Nigeria. However, more evidence is needed to explain the regional variations in the rates and odds of EIBF found in this study. Hence, we recommend that future studies further explore the regional differences in the rates and factors associated with EIBF in rural and urban Nigeria. Also, further studies are needed to investigate the causal relationship between the factors found to be associated with EIBF in rural and urban Nigeria.

## Abbreviations

AOR: Adjusted odds ratio
EIBF: Early initiation of breastfeeding
NDHS: Nigeria demographic and health survey
SPSS: Statistical package for social sciences
UHC: Universal Health Coverage
WHO: World Health Organization
